# Exploration of the carcinogenetic and immune role of CHK1 in human cancer

**DOI:** 10.7150/jca.93930

**Published:** 2024-09-16

**Authors:** Jian Zhou, Ziyi Wu, Dilihumaer Aili, Lu Wang, Tang Liu

**Affiliations:** 1 Department of Orthopedics, The Second Xiangya Hospital of Central South University, Changsha, China.; 2 Department of Orthopedic Surgery, Affiliated Hospital of Traditional Chinese Medicine, Xinjiang Medical University, Ürümqi, China.

**Keywords:** CHK1, Pan-cancer, Clinicopathological features, Prognosis, Immune infiltration.

## Abstract

**Background:** Previous study indicated that CHK1 was important for repairing DNA damage and cell cycle regulation.

**Aims:** To investigate the association of Checkpoint kinase 1 (CHK1) expression with clinicopathological features, prognosis, and immune infiltration in cancer.

**Methods:** Several databases were searched for relevant publications to conduct a meta-analysis to reveal the association between CHK1 and clinicopathological features of cancer. TIMER and GEPIA datasets were utilized to explore the differential expression of CHK1 of tumors. GEPIA and Kaplan-Meier Plotter databases were adopted to detect the prognostic significance of CHK1 in tumor. TIMER and cBioPortal databases were used for the analysis regarding immune infiltration and mutation respectively.

**Results:** We found that CHK1 expression was significantly associated with low differentiation (OR=3.94, 95% CI: 2.73-5.67, P<0.05), advanced stage (OR=3.20, 95% CI: 2.30-4.44, P<0.05), vascular infiltration (OR=3.24, 95% CI: 2.18-4.82, P<0.05) and lymph node metastasis (OR=3.55, 95% CI: 2.62-4.82, P<0.05) of various cancers. CHK1 was highly expressed in multiple cancers and was related to clinical stage, survival, immune infiltration in pan-cancers.

**Conclusions:** Our study found that CHK1 was significantly related to prognosis and immunological status in various cancers, suggesting that CHK1 may serve as a useful biomarker for prognosis and immune infiltration in cancer.

## Introduction

Cancer is a leading cause of morbidity and mortality worldwide 1. Although a substantial advancement has been made in the treatment of cancer, the clinical outcome of cancer patients who receive treatments is frequently unsatisfactory. This is mainly due to the lack of an early identification of cancer and thus a delayed treatment 2. Therefore, the identification of effective biomarkers to diagnose cancers at an early stage is of utmost importance 3-5.

CHK1 is expressed in human normal tissues and various cancers. CHK1 is an important effector molecule responsible for repairing DNA damage and cell cycle regulation 6. It has been demonstrated to play an important role in the regulation of cell cycle G1, S and G2/M phases. The inactivated CHK1 gene will lead to a haploid loss of CHK1. Cells that should stop proliferating or physiologically apoptotic will continue to enter the cell cycle, causing DNA damage to gradually accumulate during replication, eventually leading to cellular damage and malignant proliferation 7. The expression of CHK1 was found to be low in normal cells but to be high in some malignant tumors 8. Previous studies reported that CHK1 plays a crucial role in predicting clinical prognosis for multiple cancers. Al-Kaabi *et al.* found that CHK1 was an important biomarker that predicts chemotherapy response in patients with breast cancer 9. Svetlana *et al.* reported that high expression of CHK1 was related to poor differentiation and worse survival in primary lung adenocarcinomas 10. Ágnes *et al.* observed that elevated expression of CHK1 was associated with poor prognosis in lung cancer using Cox proportional hazards regression and Kaplan-Meier survival plots 11.

Considering the correlation between CHK1 and tumor prognosis, we performed a meta-analysis and pan-cancer analysis for the association of CHK1 expression with clinicopathological features of cancer. Moreover, several databases including TCGA, GEPIA, HPA, TIMER and STRING databases were applied to explore the association of CHK1 with prognosis, immune infiltration, and genetic mutation of tumor.

## Methods

### Study selection and inclusion/exclusion criteria

Several databases including NCBI PMC, NCBI PubMed, Springer, CNKI, Web of Science and Wanfang dataset were searched by two investigators JZ and TL independently to identify relevant publications published before July 31, 2024. The terms used in the search included: (“CHK1” or “CHEK1” or “checkpoint kinase 1”), (“tumor” or “cancer”), and (“clinical significance”).

Our inclusion criteria were: original paper with available data for extracting OR and 95% CI and pathological diagnosis to diagnose cancer. Exclusion criteria were: not an original study or lack of survival outcome or lack of biopsy used for the diagnosis of cancer.

### Data extraction and assessment of included studies

Two authors (JZ and TL) extracted and evaluated the relevant data including first author's name, years of publication, study design, country, type of sample, tumor type, case number, cases with CHK1 positive or negative expression, inclusion period, methods of detecting CHK1 positive expression. When additional information was needed, we contacted corresponding authors.

We used NOS score to assess the quality of included studies 12. The papers scored ≥ 7 stars were enrolled in this meta-analysis.

### Meta-analysis for the association between CHK1 and cancer

We used odds ratio (OR) and corresponding 95% confidence interval (95 % CI) to assess the association between CHK1 positive expression and various cancers. I^2^ statistics was adopted to assess the heterogeneity between the included studies 13. Fixed effects model was applied when I^2^ ≤ 50% 14. Otherwise, random effects model was utilized (I^2^ ≥ 50 %) 15. We conducted a sensitivity analysis to assess the robustness of our study results. Begg's funnel plots and Egger's test were adopted to assess the possibility of publication bias 16. We used STATA 12.0 software to conduct all these analyses. A two-tailed P < 0.05 was defined as statistical significance.

### Analysis of CHK1 expression

The TIMER database (http://timer.cistrome.org/) is a comprehensive resource for the systematical analysis of differential expression and immune infiltrates across diverse cancer types. In the present study, TIMER database was applied to assess and compare the expression level of CHK1 between normal tissues and various cancers.

GEPIA dataset (http://gepia.cancer-pku.cn/) is an online service including a series of cancer expression data. The GEPIA database contains 9,736 tumor samples and 8,587 normal samples from TCGA and is usually adopted to detect the data generated by the TCGA project. In this study, data from GEPIA database was used for making box plots to show the differential expression of CHK1 gene between tumor tissues and the corresponding normal tissues, under the settings of P-value cutoff = 0.05, log2FC cutoff =1. Moreover, we also plotted the violin plots to show the CHK1 expression at different pathological stages of multiple cancers via the “Pathological Stage Plot” module of GEPIA dataset.

The UALCAN portal (http://ualcan.path.uab.edu/analysis-prot. html) is a website for online analysis and mining of TCGA database, built based on PERL-CGI, javascript and css. Herein, we used UALCAN portal to explore the expression level of CHK1 protein in cancers including BRCA, KIRC, LUAD, OV and UCEC.

The Human Protein Atlas database (HPA) (https://www.proteinatlas.org) is a free database containing more than 26,000 kinds of antibodies. According to different dimensions, the HPA database is divided into three sections: Cell, Tissue and Pathology, which could show the expression of protein in normal tissues and cancer tissues. HPA was used to indicate CHK1 expression in tumor cells.

### Survival analysis

GEPIA can be used to perform differential expression analysis and patient survival analysis. Data from Kaplan-Meier Plotter Database (https://kmplot.com/analysis/index.php?p=service&cancer=pancancer_rnaseq) is usually used to assess the effect of gene expression on the survival of cancer patients. In the present study, GEPIA and Kaplan-Meier Plotter Databases were employed to detect the correlation between CHK1 and multiple survival status of pan-cancer including OS, DFS and RFS.

### Analysis of genetic alteration

The cBioPortal database (https://www.cbioportal.org/) for cancer genomics can be used for analysis visualization and download of large-scale tumor genomics datasets. This database was utilized to analyze the genetic alteration characteristics of CHK1. In the "Cancer Type Summary" module, we extracted the data of the change frequency, mutation type and copy number change (CNA) results of all TCGA tumors. The mutation site information of CHK1 was displayed in the protein structure diagram or 3D structure through the "Mutations" module. Additionally, "comparison" module was used to compare OS, DSS, DFS and PFS between cancer cases with and without CHK1 gene alterations.

### CHK1 expression and immune cells infiltration

The data of tumor-infiltrating immune cells in more than 10000 samples of 32 types of cancers from TCGA can be found in TIMER dataset. TIMER uses RNA-Seq expression profile data to detect the infiltration of six types of immune cells (B cells, CD4+ T cells, CD8+ T cells, Neutrophils, Macrophages and Dendritic cells) in tumor tissues. In this study, TIMER database was used to explore the association between the expression of CHK1 and immune cell infiltration in pan-cancer.

### CHK1 related gene enrichment analysis

The STRING (https://string-db.org/) database was used explore the CHK1 interacted genes, under the setting as Homo sapiens, low confidence (0.150), no more than 50 interactors and experimental. Similar Gene Detection module of GEPIA was used to show the top 100 CHK1 associated genes. Correlation Analysis module of GEPIA was adopted to conduct correlated analysis for SND1 and top 5 CHK1 associated genes. Moreover, Gene_Corr module of TIMER was used to indicate the heat map data for these genes. A venn diagram was adopted to conduct a cross-analysis for CHK1 interacted genes and associated genes. Furthermore, both two sets of data were used for GO and KEGG analysis. The gene list containing 150 genes was uploaded to DAVID dataset (https://david.ncifcrf.gov/) to obtain the Function annotation chart data. R language software [R-4.1.1-win] and Rstudio software [RStudio-1.4.1717] were used to visualize the results of KEGG and GO analyses including BP CC and MF. A two-tailed P <0.05 was defined as statistical significance.

## Results

### Features of enrolled publications

**Figure 1:**
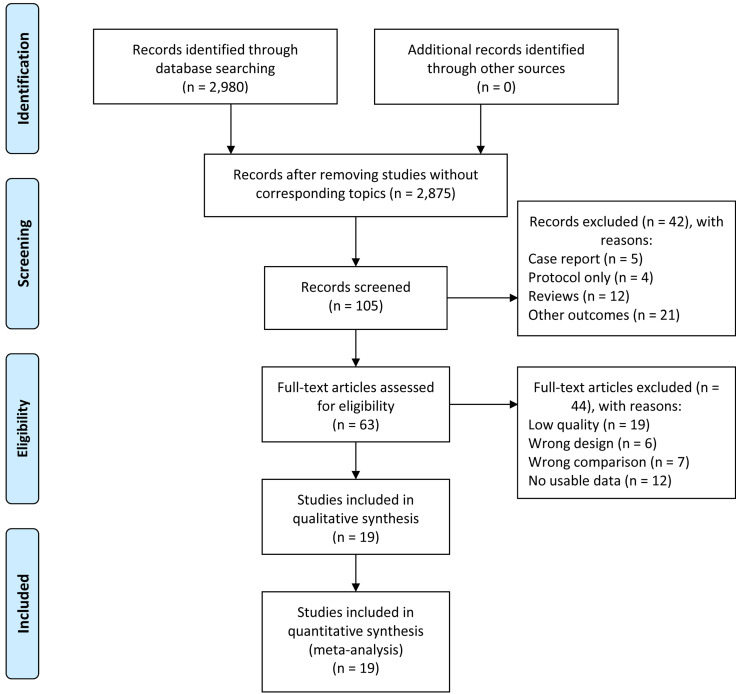
The flow chart of article selection.

The workflow of the presented study is shown in Figure 1. We obtained 2,980 articles from the initial search and screening and then excluded 2,875 articles without corresponding topic. We further excluded 42 papers because of wrong article type [case report (n=5), protocol only (n=4), review (n=12)] and lack of clinical outcomes of interest (n=21). After removing 44 literatures because of low study quality (n=19), wrong study design (n=6), wrong comparison (n=7) and no usable data (n=12), 19 articles 17-35 published from 2007 to 2019 were finally included in this meta-analysis (Figure 1).

A total of 1394 cancer patients were included in these 19 publications. The patient number in these articles range from 41 to 127, and IHC methods were used in all these publications for analyzing positive expression of CHK1. All of the studies were conducted in Asia. The NOS scores reported in these 19 publications range from 7 to 8 with an average of 7.58 (Table 1 and Table S1).

### CHK1 positive expression and clinicopathological features in cancer

**Figure 2:**
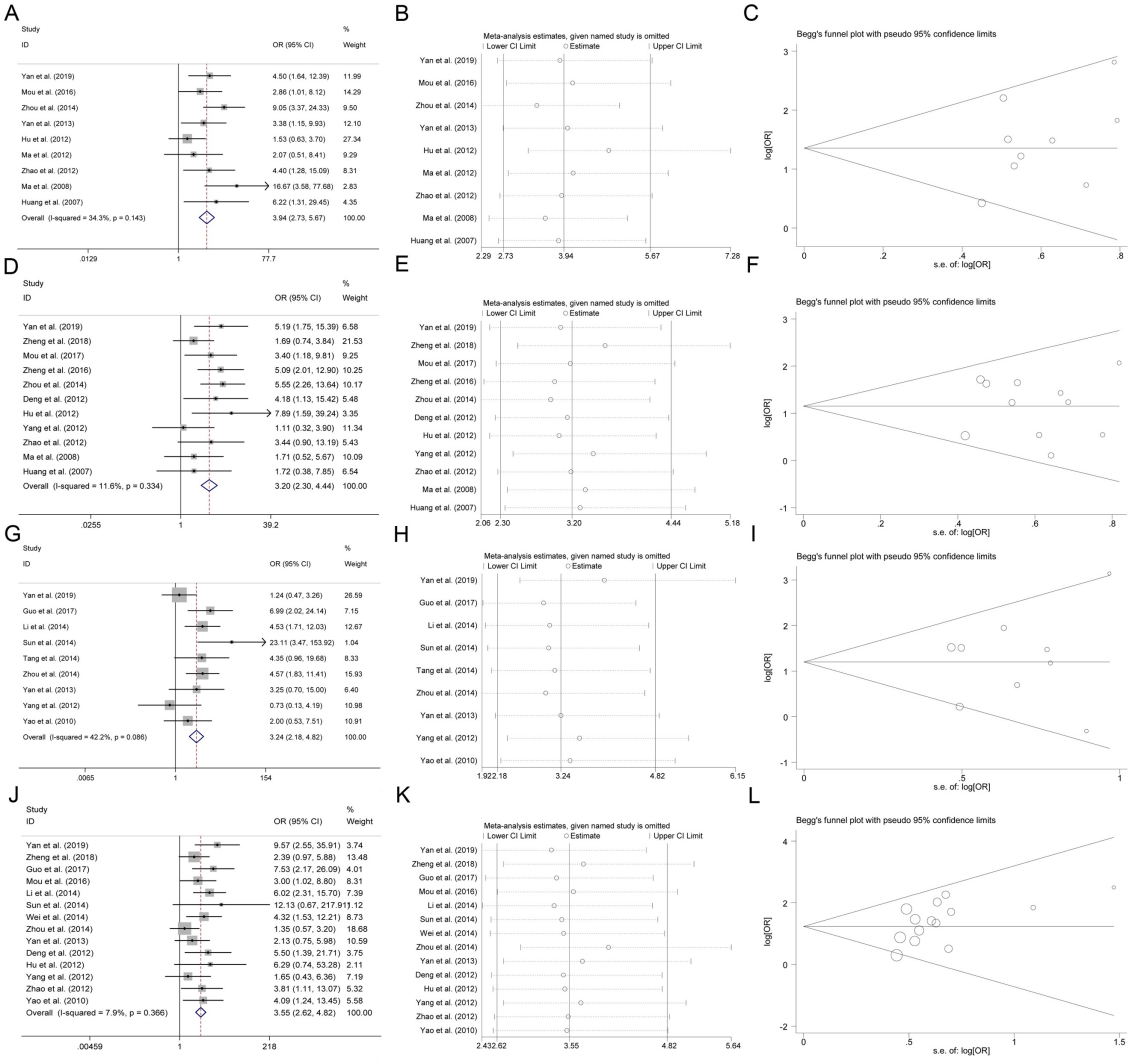
Association between CHK1 and clinicopathological feature in various cancers. **(A-C)** Forest plot, sensitivity analysis and funnel plot for association between CHK1 and cancer differentiation. **(D-F)** Forest plot, sensitivity analysis and funnel plot for association between CHK1 and cancer clinical stage. **(G-I)** Forest plot, sensitivity analysis and funnel plot for association between CHK1 and cancer vascular infiltration. **(J-L)** Forest plot, sensitivity analysis and funnel plot for association between CHK1 and cancer lymph node metastasis.

As shown in Figure 2A, we found that the expression of CHK1 was positively related to low differentiation (OR=3.94, 95% CI: 2.73-5.67, P<0.05), advanced stage (OR=3.20, 95% CI: 2.30-4.44, P<0.05), vascular infiltration (OR=3.24, 95% CI: 2.18-4.82, P<0.05), and lymph node metastasis (OR=3.55, 95% CI: 2.62-4.82, P<0.05). This result was obtained from the fixed-effect model, which was employed to pool the data given no significant between-study heterogeneity observed (I^2^<50%).

The robustness of the present analysis result was evaluated by removing a study at one time. A one-time sensitivity analysis was performed, and we found that the meta-analysis was not too dependent on a single publication, suggesting that the meta-analysis result was stable (Figure 2B). The Begg funnel plot and Egger test were conducted for evaluating the publication bias in the present meta-analysis. As shown in Figure 2C, there was no asymmetry according to the funnel plot and no publication bias was indicated by Egger test (P>0.05).

### Differential expression of CHK1 in various cancers

**Figure 3:**
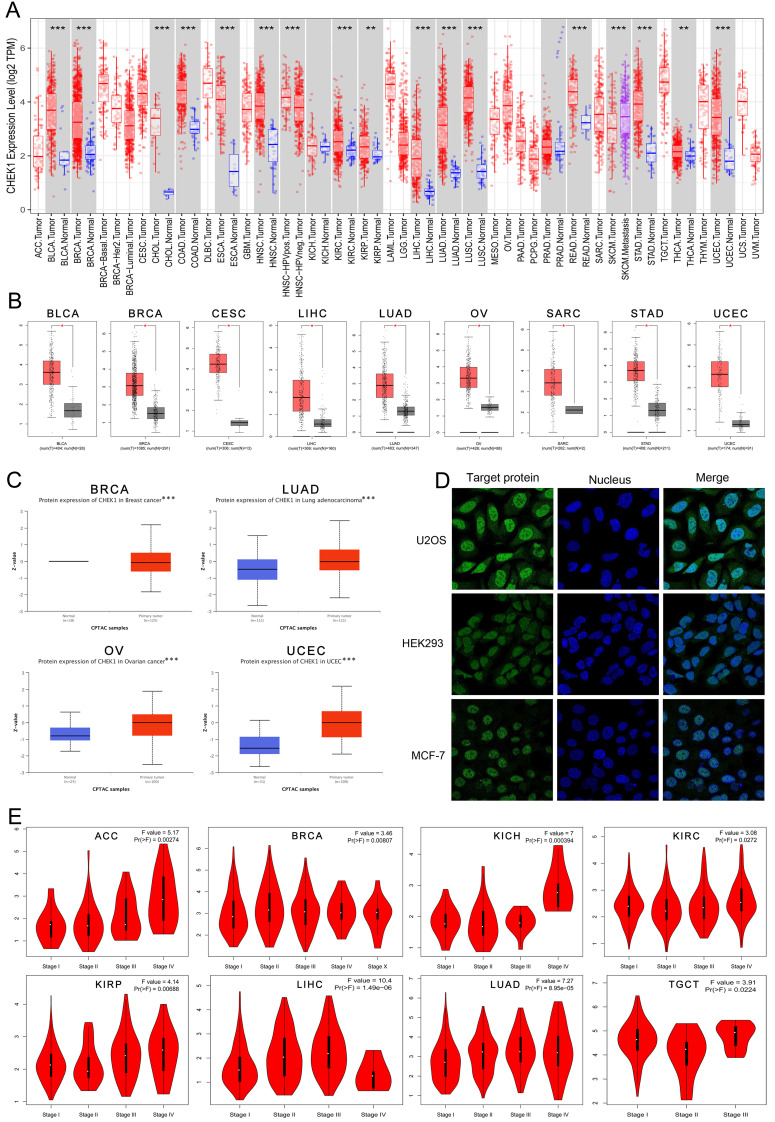
The expression of CHK1 in human cancers and clinicopathological stages. **(A)** Data from ONCOMINE database. Red cells: gene overexpression. Blue cells: decreased gene expression. Numbers: evidential frequencies. **(B)** CHK1 expression status in multiple tumors detect using TIMER dataset. ^**^P < 0.01; ^***^P <0.001. **(C)** Differential expression of CHK1 in TCGA project. ^*^P <0.05. **(D)** Differential expression of CHK1 protein in CPTAC dataset. ^***^ P < 0.001. **(E)** Correlation between CHK1 and clinical stages.

TIMER and GEPIA datasets were used to analyze the differential mRNA expression of CHK1 in multiple cancers. The data arising from TIMER shoewed that CHK1 was highly expressed in BLCA, BRCA, CHOL, COAD, ESCA, HNSC, KIRC, KIRP, LIHC, LUAD, LUSC, READ, SKCM, STAD, THCA and UCEC (Figure 3A). The data from GEPIA showed that the elevated expression of CHK1 was detected in BLCA, BRCA, CESC, LIHC, LUAD, OV, SARC, STAD, UCEC (Figure 3B). The expression of CHK1 in protein was analyzed using UALCAN portal database, and CHK1 was found that was highly expressed in BRCA, LUAD, OV and UCEC (Figure 3C). In addition, we observed the subcellular localization of CHK1 using HPA database (Figure 3D). The immunofluorescence imaging indicated that the CHK1 was mainly localized to nucleoplasm in HEK293 cell, MCF-7 cell and U2OS cell. Additionally, we found that the CHK1 expression was significantly associated with the clinical stage of ACC, BRCA, KICH, KIRC, KIRP, LIHC, LUAD and TGCT (Figure 3E).

### The prognostic significance of CHK1 in cancer

**Figure 4:**
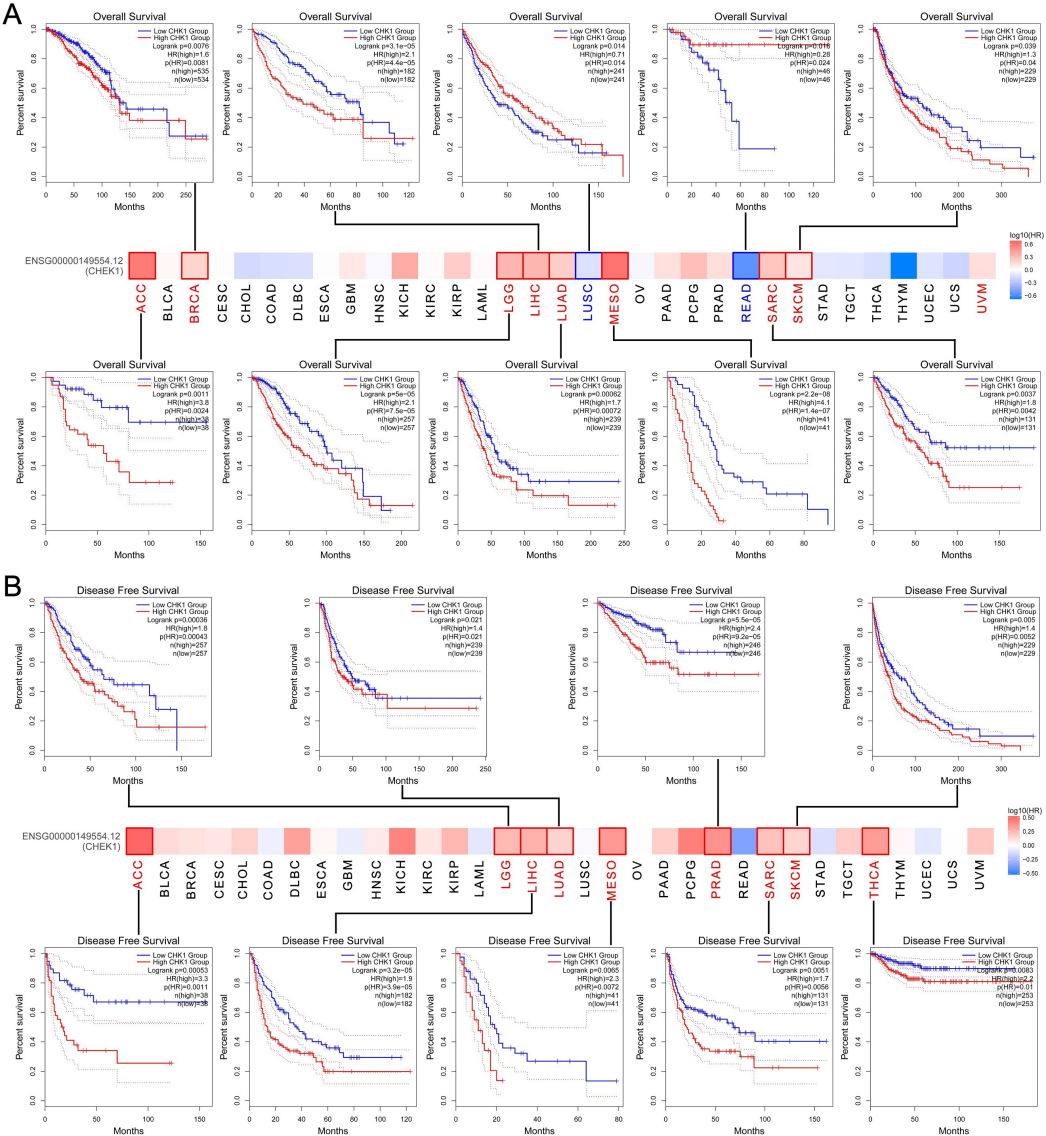
Association between CHK1 and clinical outcome of cancers in TCGA. **(A)** Overall survival, **(B)** Disease-free survival.

The GEPIA and the Kaplan-Meier Plotter databases were adopted to explore the effect of CHK1 on clinical outcomes of cancers. We found that the high expression of CHK1 was related to the worse OS in ACC, BRCA, LGG, LIHC, LUAD, MESO, SARC and SKCM, while the low expression of CHK1 was correlated with the worse OS of LUSC and READ (Figure 4A). Additionally, elevated expression of CHK1 was associated with worse DFS in ACC, LGG, LIHC, LUAD, MESO, PRAD, SARC, SKCM and THCA (Figure 4B).

**Figure 5:**
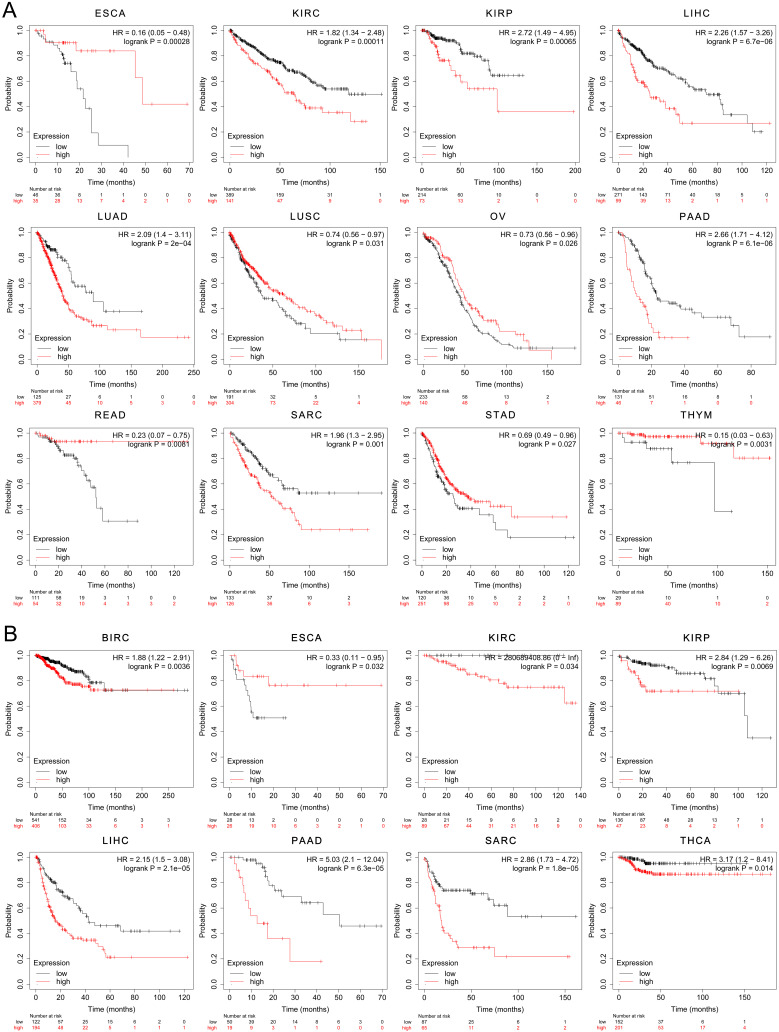
The prognostic significance of CHK1 in cancer patients. **(A)** Overall survival, **(B)** Relapse-free survival.

The results yielded from the analysis with using Kaplan-Meier Plotter dataset showed that the elevated expression of CHK1 was associated with poorer OS in KIRC, KIRP, LIHC, LUAD, PAAD and SARC. However, the low expression of CHK1 was associated with worse OS in ESCA, LUSC, OV, READ, STAD and THYM (Figure 5A). In addition, the high expression of CHK1 was associated with poorer RFS in BIRC, KIRC, KIRP, LIHC, PAAD, SARC and THCA, while the low expression of CHK1 indicated worse RFS in ESCA (Figure 5B).

### Immune infiltration level of CHK1 in cancer

We detected the relationship between CHK1 and immune infiltration of multiple cancers with TIMER database. We found that the expression of CHK1 was significantly associated with the infiltration of B cells in ACC, BRCA, BRCA-Luminal, CHOL, COAD, KIRC, LGG, LIHC, LUAD, MESO, PAAD, PCPG, STAD, TGCT, THCA, THYM and UCEC; of CD4^+^ T cells in BRCA, BRCA-Luminal, HNSC, HNSC-HPVneg, LGG, LIHC, OV, PAAD, PRAD, SARC, STAD, TGCT, THCA, THYM and UCEC; of CD8^+^ T cells in BLCA, BRCA, BRCA-Luminal, COAD, LGG, LIHC, MESO, PAAD, PCPG, READ, SARC, SKCM, SKCM-Primary, SKCM-Metastasis, TGCT, THYM and UCS; of dendritic Cell in ACC, BLCA, BRCA, BRCA-Luminal, COAD, ESCA, GBM, HNSC, HNSC-HPVneg, KIRC, LGG, LIHC, MESO, OV, PAAD, SKCM, SKCM-Primary, STAD, TGCT, THCA and THYM; of macrophage in BLCA, BRCA-Luminal, CESC, COAD, KICH, KIRC, LGG, LIHC, LUSC, MESO, OV, SARC, SKCM, SKCM-Metastasis, STAD, THCA and THYM; as well as the infiltration of neutrophil in ACC, BLCA, BRCA, RCA-Luminal, COAD, HNSC, HNSC-HPVneg, KIRC, LGG, LIHC, LUAD, MESO, OV, PCPG, READ, SKCM, SKCM-Primary, SKCM-Metastasis, THYM and UCEC (Table 2).

### Analysis of CHK1 genetic alteration

The genetic alteration of CHK1 in cancer samples in the TCGA cohorts was analyzed using cBioPortal database. The highest frequency of the alteration of CHK1 (more than 8%) was observed in patients with TGCT and UVM with “deep deletion”.

**Figure 6:**
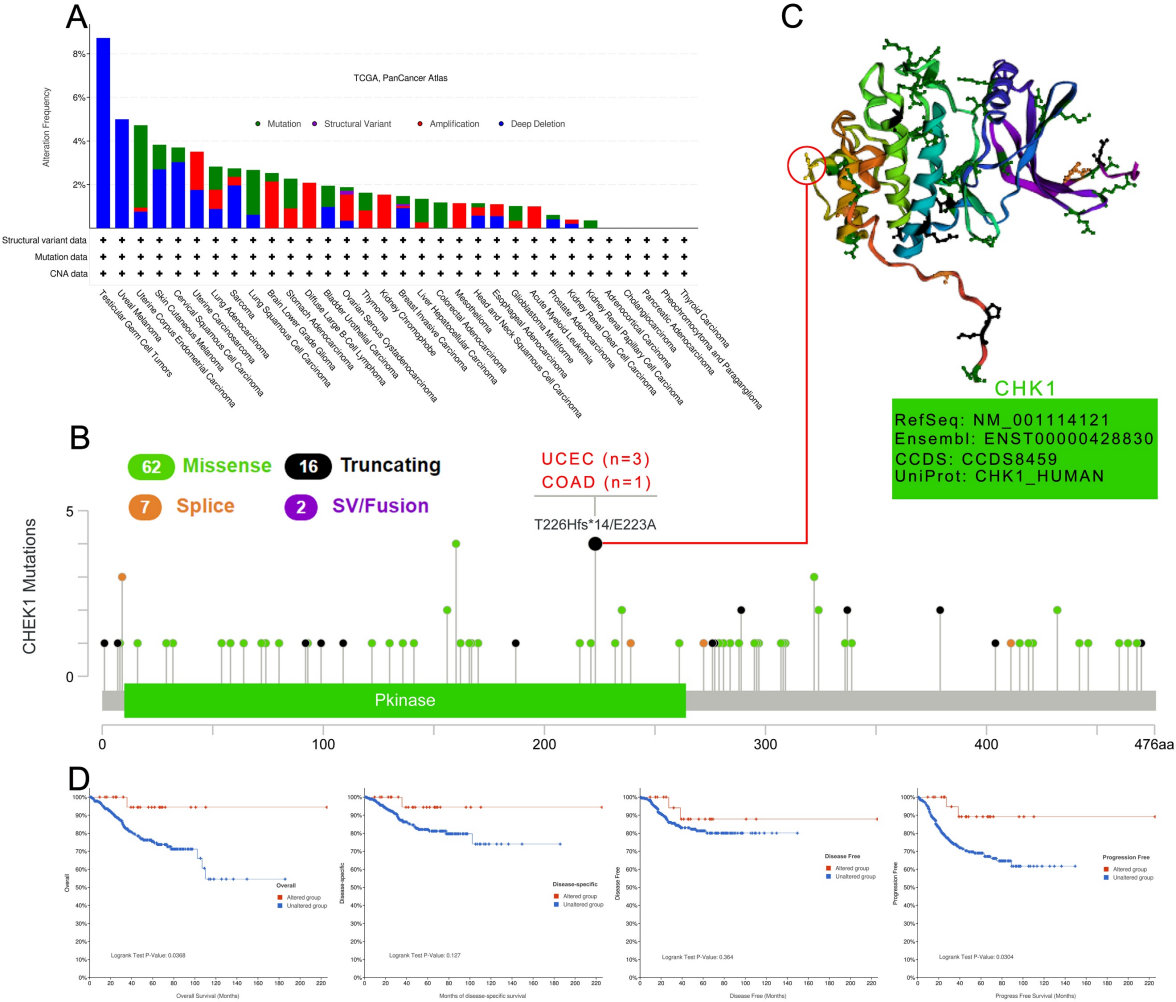
Mutation patterns of CHK1 in different cancer of TCGA. **(A)** Mutation type, **(B)** Mutation site, **(C)** The 3D structure of mutation site with the highest alteration frequency, **(D)** Association between mutation status and overall survival, disease-specific survival, disease-free survival, and progression-free survival in UCEC.

The “mutation” type was the main type of UCEC cases with an alteration frequency ranging from 4%-5% (Figure 6A). We then analyzed the types, sites and case number of CHK1 genetic alteration. According to Figure 6B, we observed that missense mutation of CHK1 was the primary type of genetic alteration. T226Hfs*14/E223A alteration was detected in the Pkinase domain with 3 cases of UCEC (T226Hfs*14, FS del) and 1 case of COAD (E223, missense) (Figure 6B). The 3D structure of the T226 site in CHK1 was shown in Figure 6C. In the analysis for assessing the relationship between CHK1 genetic alteration and prognosis of UCEC, we found that UCEC cases with altered CHK1 showed better OS (P = 0.037) and progression-free survival (P=0.030), but not better disease-specific survival (P=0.127) and DFS (P=0.364), compared to the cases without genetic alteration in CHK1 (Figure 6D).

### CHK1 associated genes enrichment analysis

**Figure 7:**
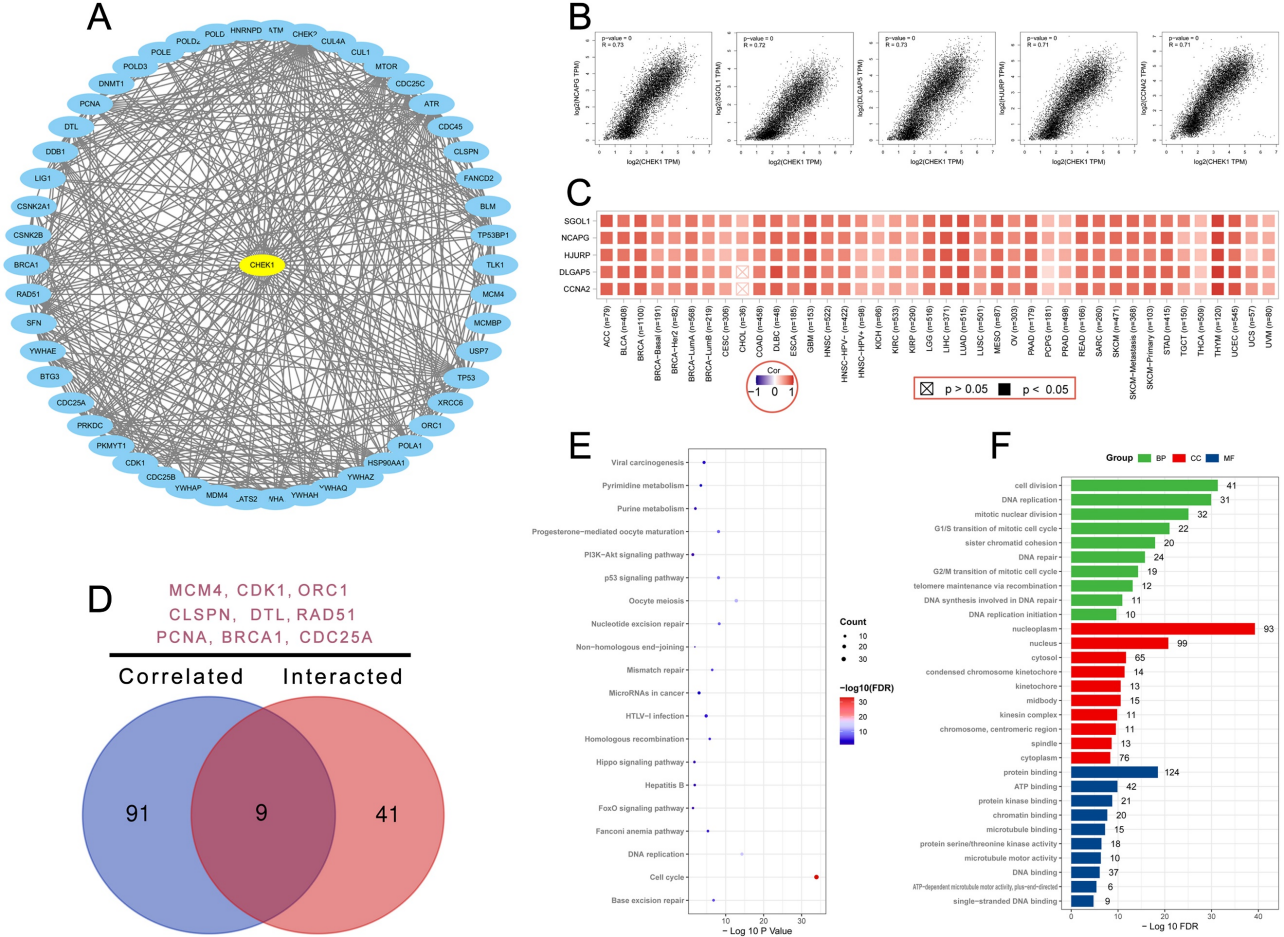
Enrichment analysis of CHK1 interacted or associated genes. **(A)** Experimentally determined interacted genes of CHK1. **(B)** Top 5 CHK1 associated genes in TCGA projects. **(C)** The corresponding heatmap map for correlation between CHK1 and top 5 related genes in various cancers. **(D)** An intersection analysis of CHK1 interacted and associated genes. **(E)** KEGG pathway analysis of CHK1 interacted or associated genes. **(F)** GO analysis of CHK1 interacted or associated genes.

STRING and GEPIA datasets were used to explore the targeting CHK1 binding proteins and CHK1 expression related genes. Fifty CHK1 binding proteins supported by experimental evidence were obtained using STRING database and the interaction network of these proteins was shown in Figure 7A. Additionally, we used GEPIA to obtain the top 100 CHK1 associated genes. The results revealed that the CHK1 was related to non-SMC condensin I complex, subunit G (NCAPG) (R=0.73, P<0.05), shugoshin 1 (SGOL1) (R=0.72, P<0.05), DLG associated protein 5 (DLGAP5) (R=0.73, P<0.05), holliday junction recognition protein (HJURP) (R=0.71, P<0.05) and cyclin A2 (CCNA2) (R=0.71, P<0.05) (Figure 7B). The heatmap data indicated a significant association between CHK1 and these 5 genes cancer (Figure 7C).

A venn diagram was plotted to delineate the common genes including minichromosome maintenance complex component 4 (MCM4), cyclin dependent kinase 1 (CDK1), origin recognition complex subunit 1 (ORC1), claspin (CLSPN), denticleless E3 ubiquitin protein ligase homolog (DTL), RAD51 recombinase (RAD51), proliferating cell nuclear antigen (PCNA), breast cancer susceptibility protein-1 (BRCA1) and cell division cycle 25A (CDC25A) in these two groups (Figure 7D). The KEGG analysis of these two groups revealed that cell cycle and DNA replication may mediate the effect of CHK1 on tumorgenesis (Figure 7E). Result of GO analysis showed that these genes were mainly enriched in cell division for BP, nucleoplasm for CC and protein binding for MF (Figure 7F).

## Discussion

There is increasing epidemiological evidence showing that cancer has surpassed cardiovascular and cerebrovascular diseases becoming as the leading cause of death in some countries 36. Although great advances have been made in promoting cancer diagnosis and treatment, the clinical prognosis and consequence in cancer patients are commonly undesirable. A very important reason is that most cancer patients are already at an advanced stage when they are diagnosed, and the therapeutic response is poor at most times 37-39. Therefore, finding biomarkers that can effectively assist clinicians in diagnosing cancer at the early stage will have a significant clinical impact.

Studies have found that cell cycle checkpoints play an important regulatory role in regulating, detecting, and maintaining cell genome stability. When the cells undergo abnormal processes such as DNA damage or replication obstruction, the regulatory mechanism involved in CHK1 is detected and activated in time. 40. CHK1 is one of the transduction factors that exist in cell cycle checkpoints in eukaryotes. It is an evolutionarily conserved serine/threonine protein kinase. It mainly acts on the regulation of cell cycle G1, S and G2/M phases 40. Current studies have found that the high expression of CHK1 is closely related to the prognosis of multiple tumors such as breast cancer, gastric cancer, oral cancer, and liver cancer 26, 28, 30, 32. The ataxia telangiectasia mutated (ATM) and ATM and Rad3-related (ATR) protein kinases exert cell cycle delay by phosphorylating checkpoint kinase 1 (CHK1) 41. ATM and ATR can phosphorylate Ser317 and Ser345 sites of CHK1. Ser280 and Ser296 sites of CHK1 can also undergo phosphorylation after DNA damage. CHK1 activated by phosphorylation can inactivate cdc25C by phosphorylating the Ser216 site of cdc25C, thus blocking the activation of cdc2 and preventing cells from entering the mitotic process 42.

Meta-analysis was a quantitative method that could synthesize the results from studies with a same research aim 43. In the first step of our study, we conducted a meta-analysis to reveal the effect of CHK1 expression on cancer. A total of 19 publications with 1394 cancer patients were included to assess the relationship between CHK1 and tumor. We observed that expression of CHK1 was positively related to low differentiation, advanced stage, vascular infiltration and lymph node metastasis. Sensitivity analysis indicated the conclusion from this meta-analysis was stable and no publication bias was reported. Specifically, we observed that CHK1 was related to differentiation in colorectal cancer, gallbladder cancer, gastric cancer, endometrial cancer, and cervical carcinoma, but not in clear cell renal cell carcinomas and hepatocellular carcinoma. CHK1 was related to advanced stages in colorectal cancer, gallbladder cancer, bladder urothelial carcinoma, gastric cancer, tongue squamous cell carcinoma, and clear cell renal cell carcinomas, but not in breast cancer, endometrial cancer, and cervical carcinoma. CHK1 was related to vascular infiltration in gastric cancer and esophageal squamous cell carcinoma, but not in colorectal cancer, hepatocellular carcinoma, or breast cancer. CHK1 was related to lymph node metastasis in colorectal cancer, gastric cancer, gallbladder cancer, esophageal squamous cell carcinoma, cervical carcinoma, and tongue squamous cell carcinoma, but not in bladder urothelial carcinoma, clear cell renal cell carcinomas, or breast cancer.

Considering the effect of CHK1 on clinicopathological characteristics of cancer, we further used several databases including TCGA, GEPIA, Kaplan-Meier Plotter, cBioPortal, STRING and DAVID datasets combined with Rstudio software to understand the association between CHK1 and the prognosis, immune infiltration and mutation of cancers. According to the results from TIMER and GEPIA, CHK1 was significantly highly expressed in various types of cancers including BLCA, BRCA, CESC, KIRC, LIHC, LUAD, OV, SARC, STAD, UCEC. etc. The high expression of CHK1 in multiple tumors showed that CHK1 may be biologically involved in the development and progression of different types of cancers. Furthermore, the association of CHK1 with clinical stage and clinical outcome of cancers was analyzed using GEPIA and Kaplan-Meier Plotter. The results revealed that the elevated expression of CHK1 was related to worse outcomes in multiple cancers, suggesting that CHK1 was a useful biomarker for predicting the prognosis of tumor.

The progression of multiple cancers can be affected by the tumor microenvironment. Immune cells of tumor microenvironment have been indicated to affect the activities in tumor. The role of CHK1 in tumor immunity involves many aspects, including enhancing immune surveillance, regulating immune escape mechanisms, and combined immunotherapy. DNA damage response (DDR) can enhance the immunogenicity of tumor cells, making them more easily recognized and attacked by the immune system. As a key regulator of DDR, CHK1 plays an important role in this process. Studies have shown that CHK1 inhibition can increase the accumulation of DNA damage in tumor cells, thereby inducing more immunogenic antigen expression 44. Additionally, tumor cells evade immune system surveillance through multiple mechanisms, and CHK1 is also involved in these immune evasion mechanisms. For example, CHK1 inhibitors can enhance the sensitivity of tumor cells to the immune system and reduce their ability to escape 45. This enhanced immune recognition can be achieved by increasing the presentation of tumor antigens or changing the expression of immunosuppressive molecules on the surface of tumor cells. Moreover, CHK1 inhibitors combined with immune checkpoint inhibitors (such as PD-1/PD-L1 inhibitors) have been shown to significantly enhance anti-tumor immune responses. CHK1 inhibition can improve the efficacy of immune checkpoint inhibitors by increasing DNA damage in tumor cells 46. This combination therapy is designed to increase the effectiveness of treatment by hitting tumor cells twice.

According to the results of TIMER database, we observed that CHK1 were significantly associated with the immune infiltration in various cancers including ACC, BRCA, BRCA-Luminal, CHOL, COAD, KIRC, LGG, LIHC, LUAD, MESO, PAAD, PCPG, STAD, TGCT, THCA, THYM and UCEC, etc., suggesting the predictive role of CHK1 in the immune status of multiple cancers. All of these data can provide useful information to inform the development of immune therapies in cancer. Gene mutations are responsible for tumorigenesis 47. In our study, the cBioPortal database was adopted to analyze the mutation sites of CHK1. The results showed that the highest alteration frequency of CHK1 was deep deletion and UCEC cases with altered CHK1 had better OS and progression-free survival, which suggested that the mutation in T226Hfs*14 site may be related to the progression of UCEC.

STRING and GEPIA datasets were used to explore the targeting CHK1 binding proteins and CHK1 expression related genes. Nine core genes including MCM4, CDK1, ORC1, CLSPN, DTL, RAD51, PCNA, BRCA1 and CDC25A were found to be as the interacted and associated genes of CHK1. CHK1 phosphorylates and regulates MCM4, part of the MCM complex involved in DNA replication initiation and elongation 48. CHK1 inhibits CDK1 activation, delaying the transition from G2 to M phase in response to DNA damage 49. CHK1 may interact with ORC1 to regulate the formation of the pre-replication complex to ensure proper replication origin firing and timing, critical for accurate DNA replication 50. CHK1 activation is facilitated by Claspin, acting as an adaptor protein and regulates DTL, involved in ubiquitination and degradation of various proteins 51. CHK1 modulates RAD51 activity, essential for homologous recombination repair of DNA double-strand breaks 52. CHK1 can phosphorylate and regulate PCNA, a critical component of the DNA replication and repair machinery 53. CHK1 interacts with BRCA1, enhancing its role in DNA damage repair and checkpoint control 54. CHK1 phosphorylates and inhibits CDC25A, preventing premature activation of CDK1 and S-phase progression 55.

Enrichment analysis showed that CHK1 interacted or associated genes were mainly enriched in cell cycle and DNA replication for KEGG analysis. Both of cell cycle and DNA replication pathway contributed significantly for tumorigenesis 56, 57. CHK1 and interacted or associated genes may be related to cancer pathogenesis. GO analysis indicated that CHK1 interacted or associated genes were primarily enriched in cell division for BP, nucleoplasm for CC and protein binding for MF. Cell division and tumorigenesis are closely related. In order to replace aging and worn-out cells, the body mainly uses a process called mitosis to divide the cell into two. When the cell is about to start dividing, it will start copying its own DNA to ensure that every daughter cell can get intact DNA. In this process, chromosomes must be accurately allocated to the progeny cells. If the DNA copy in a cell is incomplete or the DNA is damaged, it will lead to genetic disorders and diseases such as cancer 58.

Our research compiled data from multiple independent studies and found that CHK1 plays a significant role in various functions, including the cell cycle and immune environment. Through pan-cancer analysis, this finding has broader applicability, providing reliable molecular markers and novel therapeutic targets for cancer treatment. This work offers new insights into the role of CHK1 in tumor development and therapy. However, there were several limitations in this study. First, although we analyzed the correlation between CHK1 and clinicopathological features in multiple tumors, we could not explore this relationship in all cancers. Second, we didn't detect if chemotherapy or radiations affect the expression of CHK1 in cancers.

Third, all 19 studies used in our meta-analysis were conducted in China. This lack of wide representation may limit the generalizability of our findings to other populations.

## Conclusion

In this study, we used a meta-analysis to reveal the relationship between CHK1 expression and clinicopathological characteristics of cancer. Furthermore, the pan-cancer analysis of CHK1 showed the association between CHK1 and prognosis, immune infiltration, and tumor mutational burden in various cancers. All these results provided the landscape of comprehensive features of CHK1 in multiple tumors. Our findings suggested that CHK1 could be used as a novel prognostic biomarker for cancers.

## Supplementary Material

Supplementary table 1: Qualitative assessment of included study.

## Figures and Tables

**Table 1:** Characteristics of 19 studies for this meta-analysis.

No.	First author	Year	Study design	Country	Type of sample	Tumor type	Cases	CHK1 (P/N)	Inclusion period	Method	NOS score
1	Yan *et al.*	2019	Retrospective	China	Tissue	Colorectal cancer	78	53/25	2007-2011	IHC	7
2	Zheng *et al.*	2018	Prospective	China	Tissue	Bladder urothelial carcinoma	110	81/29	2009-2014	IHC	8
3	Guo *et al.*	2017	Retrospective	China	Tissue	Gastric cancer	80	63/17	2014-2015	IHC	8
4	Mou *et al.*	2016	Prospective	China	Tissue	Gallbladder cancer	64	39/25	2009-2012	IHC	8
5	Zheng *et al.*	2016	Prospective	China	Tissue	Bladder urothelial carcinoma	104	71/33	2009-2014	IHC	8
6	Li *et al.*	2014	Prospective	China	Tissue	Esophageal squamous cell carcinoma	96	65/31	2009-2010	IHC	7
7	Sun *et al.*	2014	Retrospective	China	Tissue	Esophageal squamous cell carcinoma	65	55/10	2006-2006	IHC	8
8	Tang *et al.*	2014	Retrospective	China	Tissue	Hepatocellular carcinoma	127	102/45	2005-2010	IHC	8
9	Wei *et al.*	2014	Retrospective	China	Tissue	Cervical carcinoma	121	80/41	2009-2013	IHC	8
10	Zhou *et al.*	2014	Retrospective	China	Tissue	Gastric cancer	97	50/47	2010-2013	IHC	7
11	Yan *et al.*	2013	Retrospective	China	Tissue	Gastric cancer	67	42/25	2000-2010	IHC	7
12	Deng *et al.*	2012	Retrospective	China	Tissue	Tongue squamous cell carcinoma	45	24/21	1996-2007	IHC	7
13	Hu *et al.*	2012	Prospective	China	Tissue	Clear cell renal cell carcinomas	56	39/17	2011-2011	IHC	8
14	Ma *et al.*	2012	Retrospective	China	Tissue	Hepatocellular carcinoma	41	30/11	-	IHC	8
15	Yang *et al.*	2012	Retrospective	China	Tissue	Breast cancer	47	32/15	2003-2005	IHC	7
16	Zhao *et al.*	2012	Prospective	China	Tissue	Gastric cancer	50	33/17	2007-2010	IHC	8
17	Yao *et al.*	2010	Retrospective	China	Tissue	Gastric cancer	59	35/24	-	IHC	7
18	Ma *et al.*	2008	Retrospective	China	Tissue	Endometrial cancer	44	21/23	2000-2002	IHC	8
19	Huang *et al.*	2007	Retrospective	China	Tissue	Cervical carcinoma	43	33/10	200-2002	IHC	7

**Table 2:** Association between expression of CHK1 and immune infiltration of pan-cancer by TIMER database. ^*^ P<0.05.

Cancer	Purity		B Cell		CD4+ T Cell		CD8+ T Cell		Dendritic Cell		Macrophage		Neutrophil	
	cor	p	cor	p	cor	p	cor	p	cor	p	cor	p	cor	p
ACC	0.21	0.08	0.49	0.00^*^	0.05	0.69	0.05	0.66	0.43	0.00^*^	0.11	0.38	0.27	0.02^*^
BLCA	-0.01	0.87	0.00	0.99	0.00	0.93	0.32	0.00^*^	0.34	0.00^*^	0.16	0.00^*^	0.19	0.00^*^
BRCA	0.15	0.00^*^	0.28	0.00^*^	0.11	0.00^*^	0.13	0.00^*^	0.22	0.00^*^	0.00	0.99	0.24	0.00^*^
BRCA-Basal	0.13	0.15	0.07	0.45	0.12	0.19	0.09	0.31	0.13	0.18	0.14	0.13	0.15	0.11
BRCA-Her2	0.11	0.43	-0.09	0.51	-0.01	0.91	0.00	0.99	0.05	0.71	0.10	0.45	0.20	0.14
BRCA-Luminal	0.24	0.00^*^	0.22	0.00^*^	0.10	0.02^*^	0.12	0.00^*^	0.17	0.00^*^	0.10	0.03^*^	0.18	0.00^*^
CESC	0.10	0.11	-0.07	0.28	-0.01	0.88	-0.04	0.55	-0.06	0.29	-0.14	0.02^*^	0.02	0.75
CHOL	-0.21	0.22	-0.36	0.03^*^	-0.27	0.12	0.08	0.67	-0.28	0.10	0.02	0.91	0.14	0.44
COAD	0.08	0.12	0.19	0.00^*^	0.07	0.16	0.18	0.00^*^	0.19	0.00^*^	0.12	0.01^*^	0.23	0.00^*^
DLBC	0.25	0.11	0.44	0.06	-0.41	0.07	-0.09	0.69	0.20	0.38	0.00	1.00	-0.08	0.73
ESCA	0.22	0.00^*^	0.13	0.09	-0.09	0.21	-0.05	0.51	-0.32	0.00^*^	0.05	0.52	-0.14	0.05
GBM	0.39	0.00^*^	-0.10	0.05	-0.05	0.35	-0.05	0.32	0.14	0.00^*^	-0.09	0.07	0.01	0.83
HNSC	0.18	0.00^*^	0.05	0.27	0.20	0.00^*^	0.07	0.15	0.19	0.00^*^	0.04	0.38	0.20	0.00^*^
HNSC-HPVpos	0.26	0.01^*^	0.07	0.53	0.14	0.22	0.12	0.31	0.07	0.54	-0.14	0.20	0.08	0.46
HNSC-HPVneg	0.12	0.01^*^	-0.01	0.86	0.20	0.00^*^	0.01	0.81	0.18	0.00^*^	0.05	0.33	0.20	0.00^*^
KICH	-0.03	0.82	0.17	0.18	0.18	0.16	0.12	0.33	0.11	0.36	0.31	0.01^*^	-0.08	0.50
KIRC	0.01	0.81	0.22	0.00^*^	0.09	0.07	0.09	0.05	0.27	0.00^*^	0.22	0.00^*^	0.27	0.00^*^
KIRP	0.21	0.00^*^	0.06	0.31	-0.10	0.10	0.03	0.63	0.04	0.51	-0.01	0.90	-0.08	0.18
LGG	0.20	0.00^*^	0.21	0.00^*^	0.17	0.00^*^	0.16	0.00^*^	0.24	0.00^*^	0.20	0.00^*^	0.15	0.00^*^
LIHC	0.18	0.00^*^	0.44	0.00^*^	0.30	0.00^*^	0.35	0.00^*^	0.47	0.00^*^	0.42	0.00^*^	0.38	0.00^*^
LUAD	0.00	0.98	-0.18	0.00^*^	-0.06	0.17	0.07	0.11	0.02	0.64	-0.02	0.61	0.15	0.00^*^
LUSC	0.29	0.00^*^	0.01	0.86	0.04	0.43	0.04	0.40	0.01	0.84	-0.12	0.01^*^	0.04	0.40
MESO	-0.08	0.49	0.29	0.01^*^	0.10	0.35	0.24	0.03^*^	0.36	0.00^*^	0.23	0.04^*^	-0.27	0.01^*^
OV	0.10	0.03^*^	0.04	0.42	0.12	0.01^*^	-0.07	0.14	0.14	0.00^*^	0.09	0.04^*^	0.16	0.00^*^
PAAD	0.02	0.76	0.16	0.03^*^	-0.19	0.01^*^	0.18	0.02^*^	0.23	0.00^*^	0.10	0.20	0.09	0.25
PCPG	0.24	0.00^*^	0.17	0.03^*^	0.03	0.75	-0.38	0.00^*^	0.01	0.85	-0.06	0.47	-0.24	0.00^*^
PRAD	0.21	0.00^*^	0.01	0.89	0.12	0.01^*^	-0.08	0.11	0.00	0.98	0.03	0.56	0.03	0.54
READ	0.04	0.62	0.14	0.10	-0.12	0.17	0.22	0.01^*^	0.12	0.17	-0.05	0.56	0.23	0.01^*^
SARC	0.29	0.00^*^	0.13	0.05	-0.33	0.00^*^	0.16	0.01^*^	-0.02	0.77	-0.20	0.00^*^	-0.12	0.07
SKCM	0.10	0.03^*^	0.08	0.11	0.06	0.24	0.22	0.00^*^	0.16	0.00^*^	0.18	0.00^*^	0.34	0.00^*^
SKCM-Primary	0.18	0.07	0.17	0.09	-0.05	0.59	0.34	0.00^*^	0.22	0.03^*^	0.16	0.12	0.44	0.00^*^
SKCM-Metastasis	0.08	0.14	0.00	0.96	0.03	0.57	0.14	0.01^*^	0.07	0.20	0.12	0.02^*^	0.26	0.00^*^
STAD	0.13	0.01^*^	-0.19	0.00^*^	-0.20	0.00^*^	-0.09	0.07	-0.13	0.01^*^	-0.30	0.00^*^	-0.08	0.14
TGCT	-0.05	0.52	0.19	0.02^*^	-0.17	0.03^*^	0.31	0.00^*^	0.36	0.00^*^	-0.12	0.16	-0.07	0.37
THCA	0.02	0.73	0.28	0.00^*^	0.14	0.00^*^	0.04	0.33	0.19	0.00^*^	0.12	0.01^*^	0.07	0.11
THYM	-0.16	0.10	0.79	0.00^*^	0.72	0.00^*^	0.63	0.00^*^	0.81	0.00^*^	0.62	0.00^*^	-0.22	0.02^*^
UCEC	0.05	0.40	-0.17	0.00^*^	-0.15	0.01^*^	0.11	0.07	-0.02	0.70	-0.11	0.05	0.29	0.00^*^
UCS	-0.05	0.72	0.21	0.12	-0.22	0.11	0.33	0.02^*^	0.14	0.31	0.13	0.37	0.12	0.38
UVM	0.22	0.05	0.12	0.29	-0.22	0.05	0.08	0.48	-0.02	0.86	-0.16	0.20	-0.05	0.68

## Abbreviations

CHK1: Checkpoint kinase 1
TCGA: The Cancer Genome Atlas
GEPIA: Gene Expression Profiling Interactive Analysis
STRING: Search tool for the retrieval of interacting genes
CNKI: China National Knowledge Internet database
NOS: Newcastle-Ottawa quality assessment scale
OR: Odds ratio
95% CI: 95% confidence interval
FC: Fold change
TIMER: Tumor immune estimation resource
BRCA: Breast cancer
KIRC: Clear cell Renal cell carcinoma
LUAD: Lung adenocarcinoma
OV: Ovarian cancer
UCEC: Uterine corpus endometrial carcinoma
CAN: Copy number change
3D: Three-dimensional
OS: Over survival
DSS: Disease-specific survival
DFS: Disease-free survival
PFS: Progression-free survival
KEGG: Kyoto Encyclopedia of Genes and Genomes
GO: Gene Ontology
BP: Biological process
CC: Cell composition
MF: Molecular function
BLCA: Bladder Urothelial Carcinoma
CESC: Cervical squamous cell carcinoma and endocervical adenocarcinoma
COAD: Colon adenocarcinoma
ESCA: Esophageal carcinoma
STAD: Stomach adenocarcinoma
HNSC: Head and Neck squamous cell carcinoma
LUSC: Lung squamous cell carcinoma
DLBC: Lymphoid Neoplasm Diffuse Large B-cell Lymphoma
SKCM: Skin Cutaneous Melanoma
SARC: Sarcoma
CHOL: Cholangiocarcinoma
KIRP: Kidney renal papillary cell carcinoma
LIHC: Liver hepatocellular carcinoma
READ: Rectum adenocarcinoma
THCA: Thyroid carcinoma
ACC: Adrenocortical carcinoma
LGG: Brain Lower Grade Glioma
MESO: Mesothelioma
PAAD: Pancreatic adenocarcinoma
THYM: Thymoma
PCPG: Pheochromocytoma and Paraganglioma
TGCT: Testicular Germ Cell Tumors
NCAPG: Non-SMC condensin I complex, subunit G
SGOL1: Shugoshin 1
DLGAP5: DLG associated protein 5
HJURP: Holliday junction recognition protein
CCNA2: Cyclin A2
MCM4: Minichromosome maintenance complex component 4
CDK1: Cyclin dependent kinase 1
ORC1: Origin recognition complex subunit 1
CLSPN: Claspin
DTL: Denticleless E3 ubiquitin protein ligase homolog
RAD51: RAD51 recombinase
PCNA: Proliferating cell nuclear antigen
BRCA1: Breast cancer susceptibility protein-1
CDC25A: Cell division cycle 25A
