# Supplementary material for: Exploration of the carcinogenetic and immune role of CHK1 in human cancer
Source: J Cancer. 2024 Sep 16;15(18):5927–41. doi: 10.7150/jca.93930 (PMC11493005; doi:10.7150/jca.93930)
Supplement: Supplementary file 1 — Supplementary table 1: Qualitative assessment of included study. [file jcav15p5927s1.pdf]

Table S1. Qualitative assessment of included study.

Column	Entries	Study									
		1	2	3	4	5	6	7	8	9	10
Section	Is the definition adequate	☆	☆	☆	☆	☆	☆	☆	☆	☆	☆
	Representativeness of the cases	☆	☆	☆	☆	☆	☆	☆	☆	☆	☆
	Selection of controls										
	Definition of controls	☆	☆	☆	☆	☆	☆	☆	☆	☆	☆
Comparability	Comparability of cases and controls on the basis of the design and analysis	☆	☆☆	☆☆	☆☆	☆☆	☆	☆☆	☆☆	☆☆	☆
Exposure	Ascertainment of exposure	☆	☆	☆	☆	☆	☆	☆	☆	☆	☆
	Same method of ascertainment for cases and controls	☆	☆	☆	☆	☆	☆	☆	☆	☆	☆
	Non-Response rate	☆	☆	☆	☆	☆	☆	☆	☆	☆	☆
Total scores		7	8	8	8	8	7	8	8	8	7

Notes: 1. Yan et al. 2019, 2. Zheng et al. 2018, 3. Guo et al. 2017, 4. Mou et al. 2016, 5. Zheng et al. 2016, 6. Li et al. 2014, 7. Sun et al. 2014, 8. Tang et al. 2014, 9. Wei et al. 2014, 10. Zhou et al. 2014.

Column	Entries	Study								
		11	12	13	14	15	16	17	18	19
Section	Is the definition adequate	☆	☆	☆	☆	☆	☆	☆	☆	☆
	Representativeness of the cases	☆	☆	☆	☆	☆	☆	☆	☆	☆
	Selection of controls									
	Definition of controls	☆	☆	☆	☆	☆	☆	☆	☆	☆
Comparability	Comparability of cases and controls on the basis of the design and analysis	☆	☆	☆☆	☆☆	☆	☆☆	☆	☆☆	☆
Exposure	Ascertainment of exposure	☆	☆	☆	☆	☆	☆	☆	☆	☆
	Same method of ascertainment for cases and controls	☆	☆	☆	☆	☆	☆	☆	☆	☆
	Non-Response rate	☆	☆	☆	☆	☆	☆	☆	☆	☆
Total scores		7	7	8	8	7	8	7	8	7

Notes: 11. Yan et al. 2013, 12. Deng et al. 2012, 13. Hu et al. 2012, 14. Ma et al. 2012, 15. Yang et al. 2012, 16. Zhao et al. 2012, 17. Yao et al. 2010, 18. Ma et al. 2008, 19. Huang et al. 2007.
